# Currently approved imaging modalities of atrial fibrillation drivers: Are they in agreement?

**DOI:** 10.1016/j.hrcr.2023.07.022

**Published:** 2023-08-09

**Authors:** Gregory Cunn, Kristie Coleman, Stavros Mountantonakis

**Affiliations:** Department of Cardiac Electrophysiology, Northwell Health - Lenox Hill Heart & Lung, New York, New York

**Keywords:** Atrial fibrillation, Catheter ablation, Artificial intelligence, Dispersion, Noninvasive mapping, Driver

**Key Teaching Points**

• The case illustrates a promising approach combining noninvasive electrocardiographic mapping (CardioInsight; Medtronic) and real-time artificial intelligence adjudication of multipolar electrogram dispersion (VX1, Volta Medical) for treatment of a persistent atrial fibrillation (AF) patient.
• Rotational activity detected with noninvasive mapping correlates with dispersion areas detected with VX1 on the floor of the left atria.
• Artificial intelligence–based software VX1 offers an attractive solution to assist operators targeting of AF drivers.

## Introduction

The incidence and prevalence of atrial fibrillation (AF) are continuing to rise worldwide. Consequently, the management of AF represents a therapeutic challenge. While catheter ablation is commonly used for treatment of persistent AF, there remains insufficient data to identify which regions should be addressed, and debate continues over what comprises the optimal lesion set.^1^, ^2^, ^3^, ^4^ AF has been shown to be driven by localized electrical sources of fibrillatory activity, which may include localized anatomical reentries, functional rotors, or focal discharges.^5^^,^^6^ Therefore, per-operative technologies that identify driver regions are particularly relevant. Medtronic (Minneapolis, MN) acquired an electrocardiographic imaging system (CardioInsight) that reconstructs epicardial potentials, electrograms (EGMs), and isochrones from electrocardiographic body surface potentials using a multielectrode mapping vest containing 252 electrodes.^7^ Collected signals are processed using inverse solution–based algorithms and allow for building activation, voltage, isopotential, and phase maps.^8^ These maps have been shown to depict localized AF dynamics in both atria. Previously, other mapping technologies similarly provided advanced interpretation of EGM recordings. These approaches aimed at highlighting ablation-relevant atrial regions of interest, which harbor AF drivers.^9^^,^^10^ These technologies, based on a panoramic appraisal of atrial activity, obtained simultaneous EGMs from multiple atrial locations but were limited in their ability to provide a detailed evaluation of local electrical activity during AF. Recently, Seitz and colleagues^11^ described a multipolar EGM marker of AF drivers, the spatiotemporal dispersion, which is based on iterative local recordings of AF activity. Dispersion corresponds to the clustering in space of multipolar EGMs exhibiting differing morphologies.^11^ To assist operators in the real-time detection of spatiotemporal dispersion, Volta Medical developed a deep/machine learning software named VX1 (Marseille, France). VX1’s interface highlights in real time bipoles exhibiting a high statistical likelihood of the presence of dispersion. Dispersion maps are then built with the iterative tagging regions of interest on the biatrial 3D shell of the 3D mapping system. Recently, VX1 has been shown to allow for center-to-center standardization of procedural and long-term outcomes after VX1-highlighted regions have been ablated.^12^ Still, it remains unclear how VX1-mapped dispersion regions may correspond to identifiable patterns of AF dynamics such as rotors and figures-of-8. Both noninvasive electroanatomical mapping and spatiotemporal dispersion mapping, the only currently FDA-approved technologies to image rotors, are limited by their inability to image the meandering rotors characteristic of persistent AF. Therefore, this case report demonstrates an interesting clinical application of existing technologies that were shown to demonstrate a correlation in their findings. In this patient the combined approach allowed for the AI-assisted mapping of spatiotemporal dispersion in a persistent AF patient, while at the same time the electrocardiographic imaging / CardioInsight depicted localized AF dynamics with a high degree of correlation.

## Case report

A 66-year-old patient presented with a history of recurrent symptomatic persistent AF. The patient had undergone 2 prior AF ablations in 2018 and 2020. During the first ablation procedure a pulmonary vein isolation was performed. During the second ablation, the pulmonary veins (PVs) were confirmed to be isolated with antral isolation. The level of isolation for the right PVs was extended septally. Earliest activation was seen at the coronary sinus and lesions were delivered epicardially and endocardially. Early signals were noted in the right atrial appendage (RAA), and ablation within the RAA rendered AF noninducible after delivery of isoproterenol. A typical flutter line was created along the cavotricuspid isthmus and block was confirmed. The patient experienced recurrent symptomatic persistent AF, and the decision was made to proceed with a third ablation at Lenox Hill Hospital, Manhattan, NY. The patient discontinued flecainide for 2 half-lives prior to presenting for the ablation procedure.

After informed consent was obtained, noninvasive electrocardiographic mapping was first performed using the CardioInsight vest (Medtronic). The patient underwent a noncontrast computed tomography scan of the chest to provide anatomic data for mapping. She subsequently underwent an endocardial ablation as follows: (1) patient was placed under general anesthesia, (2) access was obtained in the femoral veins with ultrasound guidance, (3) a multipole catheter was placed into the coronary sinus, and (4) a single trans-septal puncture was performed under intracardiac echocardiography guidance after administration of intravenous heparin to maintain an activated clotting time >350 seconds. Esophageal temperature was monitored and a 3-dimensional cardiac mapping system (CARTO® 3; Biosense Webster, Irvine, CA) was used to guide the procedure.

**Figure 1:**
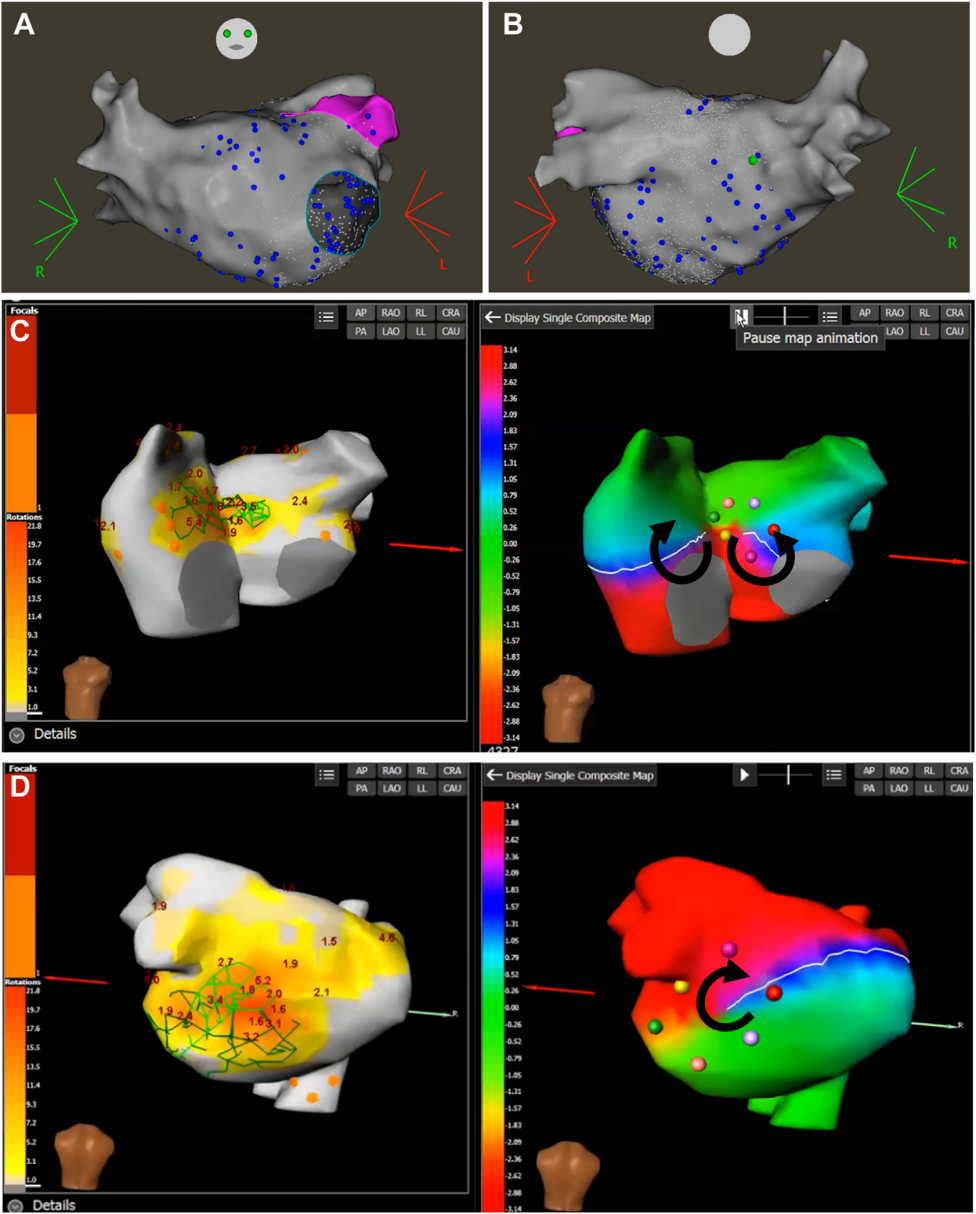
Representative electroanatomic maps with anteroposterior view (**A**) and posteroanterior view (**B**) showing dispersion sites identified by VX1 system, Volta Medical (*blue tags*). Representative pictures of 2 distinct functional reentrant patterns identified in the patient (**C, D**). Left panels correspond to electrocardiographic imaging composite maps of the patient. Right panels correspond to noninvasive activation map (CardioInsight; Medtronic) of the atria showing spontaneous atrial fibrillation drivers. Black arrows depict the sequence of activation.

**Figure 2:**
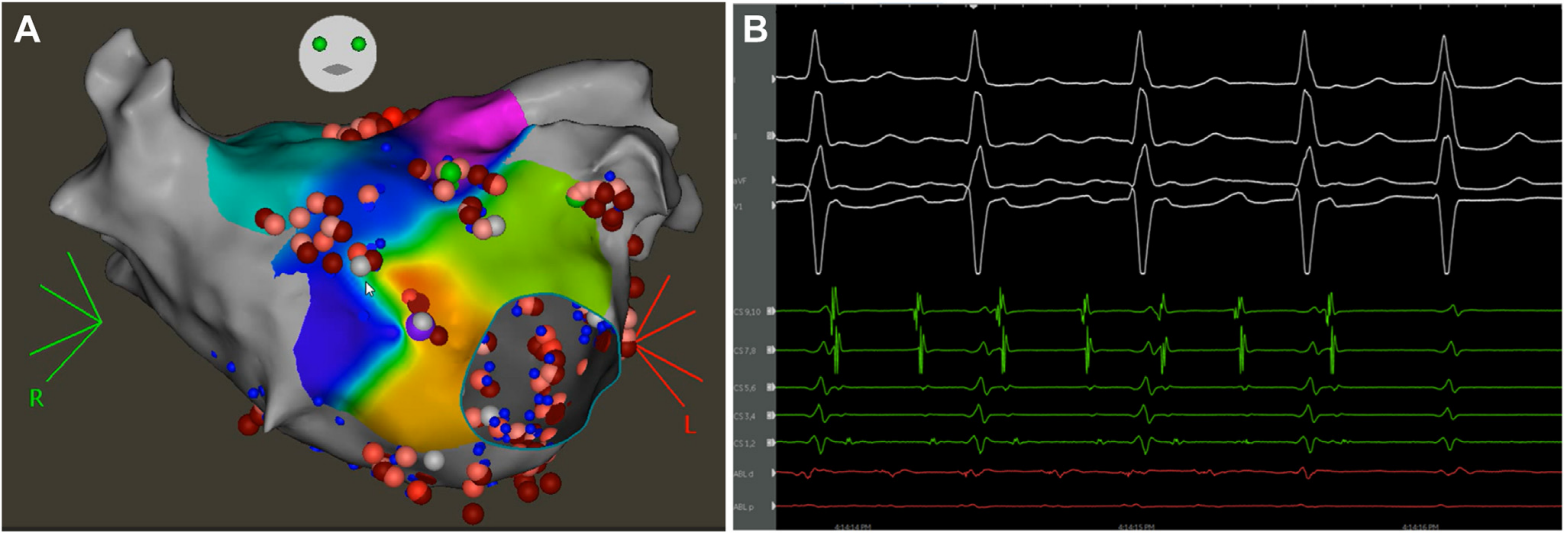
Ablation and termination. **A:** Electroanatomical maps with anteroposterior view showing ablation set. Ablation at dispersion regions led to atrial fibrillation termination. **B:** Electrograms from the ablation catheter at the site of successful ablation.

Rapid pacing from the Pentaray catheter in the left atrium induced AF. A multielectrode Pentaray catheter (Biosense Webster) was employed to map left and right atria sequentially and to provide real-time adjudications of dispersion to build biatrial dispersion maps. All 4 PVs were confirmed to be isolated with antral level of isolation. Dispersion adjudication was performed with the VX1 system (Volta Medical) and ablation at dispersion areas was performed as described previously.^12^ The dispersion regions spanned the posterior left atrium, the floor of the left atrium, and the anterior wall of the left atrium (Figure 1A and 1B). Based on the areas of dispersion marked by the VX1 system, a posterior wall isolation was performed. Subsequently, a lateral mitral isthmus line was created and additional ablation lesions were delivered epicardially in the coronary sinus. While lesions were being delivered along the anterior mitral isthmus, AF terminated to sinus rhythm, and block was confirmed along the mitral isthmus line (Figure 2).

As shown in Figure 1C and 1D, as well as in Supplemental Videos 1 and 2, 2 distinct functional reentrant patterns were identified:

(1) A multiple-rotation figure-of-8, from which counter-rotating waves emanated to both atria. The 2 rotors forming the figure-of-8 pattern were centered on 2 tagged points, 1 located near the anterior part of the septum and the other pinned on the left atrial anterior wall.
(2) A multiple rotation reentrant pattern centered on the posterior wall.

Interestingly, both pattern (1) and pattern (2) were found in the midst of a dispersion region (see Figure 1). Also, both rotational activities were found within an area of continuous complex low-amplitude EGMs and surrounded by areas of much larger-amplitude EGMs on ripple mapping (CARTO). These regions of interest were partially overlapping with low-voltage regions, albeit without an exact overlap with areas of low voltage. Rotational activity on the floor of the left atrium with noninvasive mapping (CardioInsight) correlated with dispersion areas (VX1) correlated with areas of continuous complex low-amplitude EGMs surrounded by areas of much larger-amplitude EGMs on ripple mapping (CARTO) (Figure 2). The sheaths were withdrawn into the right atrium and bidirectional block was confirmed along the cavotricuspid isthmus. Areas of dispersion were identified at the base of the RAA, additional lesions were delivered.

Recurrent AF was monitored via implantable loop recorder. The patient experienced early recurrence of AF 2 months after the ablation procedure, for which she was successfully cardioverted. The patient continued flecainide for 12 months postprocedure and remained in normal sinus rhythm. The patient experienced recurrent AF 16 months postprocedure, for which she was successfully cardioverted. Overall, the patient’s AF burden remained less than 2%. The patient is maintained on continuous oral anticoagulation.

## Discussion

We report on the case of a 66-year-old patient admitted for repeat ablation of recurrent persistent AF. We used a combination of noninvasive mapping and endocardial, high-density, artificial intelligence (AI)-enabled VX1 adjudication of multipolar EGM dispersion.

This case exemplifies the following:

(1) Persistent AF may be advantageously characterized with a combined endocardial-epicardial mapping approach, the former focused on dispersion mapping, the latter enabling visualization of AF dynamics.
(2) Dispersion may correspond to identifiable patterns of propagation known to have the ability to drive AF, such as rotors and figures-of-8.^13^ While rotor or figure-of-8 wavefronts propagate widely through both atria, dispersion mapping allows for a delineation of regions of interest, which may be considered for ablation, although the technology is still limited by its inability to image meandering rotors.

In this setting, using AF termination as procedural endpoint is supported by both a mechanism-elucidating mapping approach (CardioInsight; Medtronic) and a machine/deep learning algorithm (VX1; Volta Medical).

Previously, various mapping approaches have been employed: Topera, Carto Finder, Ablacon. Here, we combined 2 distinct EGM-based approaches: the CardioInsight noninvasive maps focusing on large patterns of propagation detected at the biatrial scale and postprocessing of signals from the time domain to the frequency domain (Hilbert transform). In contrast, the VX1 system elucidates the presence of spatial clusters of multipolar EGMs of various morphologies. The latter characteristic of VX1’s technology is unique in that EGMs adjudicated as a “cluster” by the AI engine may present as low or high voltage, fast or slow frequency, fractionated or nonfractionated. In other words, the visual cues commonly used in the electrophysiology lab to probe for relevant EGMs are not necessarily the ones that VX1 is interested in.

Beyond the simultaneous use of the CardioInsight and VX1 technologies, this case provides an opportunity to refine both systems. Conceivably, the information provided by either set of analytics could be used to “feed” future software or algorithm solutions, although both are limited by their inability to image meandering rotors. For example, it would be valuable to build a larger database of combined VX1-CardioInsight cases and evaluate whether the AI engine could predict the presence of specific patterns of propagation that developed at corresponding locations. Conversely, VX1 may provide an opportunity for analytical refinement of the CardioInsight system in allowing for an improved detection when local EGM conditions are not ideal for noninvasive recordings, such as low-voltage areas and/or previously ablated regions. More generally, the ERASE trial has recently shown that extra-PV ablation for persistent AF patients might still be relevant.^14^ Therefore, one may anticipate a renewed interest in technologies that could standardize our approach to extra-PV lesions and further develop technologies to image AF effectively.

## Conclusion

We present our case using the combined implementation of the CardioInsight system with a distinct EGM-based AI approach (VX1; Volta Medical). Data collected showed how pattern elucidation with CardioInsight advantageously complements an approach based on the analysis of multipolar EGMs in real time, although both technologies are currently limited by their inability to image meandering rotors.

## Appendix Supplementary Data

Supplemental Video 1 Multiple-rotation figure-of-8.

Supplemental Video 2 Multiple rotation reentrant pattern centered on the posterior wall.
